# Viral entry mechanisms: the role of molecular simulation in unlocking a key step in viral infections

**DOI:** 10.1002/2211-5463.13908

**Published:** 2024-10-14

**Authors:** Mariana Valério, Carolina C. Buga, Manuel N. Melo, Cláudio M. Soares, Diana Lousa

**Affiliations:** ^1^ Instituto de Tecnologia Química e Biológica Universidade Nova de Lisboa Oeiras Portugal; ^2^ Instituto de Medicina Molecular Faculdade de Medicina da Universidade de Lisboa Lisbon Portugal

**Keywords:** enveloped viruses, fusion peptide, membrane fusion, molecular simulation, receptor binding, viral entry

## Abstract

Viral infections are a major global health concern, affecting millions of people each year. Viral entry is one of the crucial stages in the infection process, but its details remain elusive. Enveloped viruses are enclosed by a lipid membrane that protects their genetic material and these viruses are linked to various human illnesses, including influenza, and COVID‐19. Due to the advancements made in the field of molecular simulation, significant progress has been made in unraveling the dynamic processes involved in viral entry of enveloped viruses. Simulation studies have provided deep insight into the function of the proteins responsible for attaching to the host receptors and promoting membrane fusion (fusion proteins), deciphering interactions between these proteins and receptors, and shedding light on the functional significance of key regions, such as the fusion peptide. These studies have already significantly contributed to our understanding of this critical aspect of viral infection and assisted the development of effective strategies to combat viral diseases and improve global health. This review focuses on the vital role of fusion proteins in facilitating the entry process of enveloped viruses and highlights the contributions of molecular simulation studies to uncover the molecular details underlying their mechanisms of action.

## Graphical abstract

This article reviews how molecular simulations can help understand the complex process of viral membrane fusion, an essential step in viral infection. Simulations can not only complement experimental techniques but also provide a deeper understanding of how viral fusion proteins interact with host cells, helping to inform the development of next‐generation antiviral treatments to combat emerging viral threats.

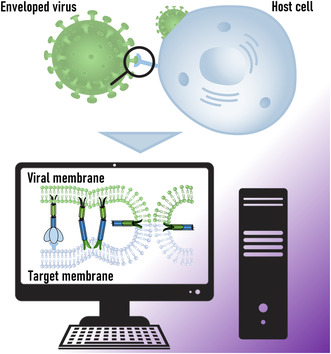

**Abbreviations**
AA: all‐atom
BH: beta‐hairpin
CG: coarse‐grain
CH: central helix
CR: connecting region
CT: cytoplasmic tail
E: envelope glycoprotein
F protein: fusion protein
FEP: free energy perturbation
FP: fusion peptide
GP: golden peptide
HA: hemagglutinin
HR1: heptad repeat 1
HR2: heptad repeat 2
IFP: influenza fusion peptide
MD: molecular dynamics
PIFP: parainfluenza fusion peptide
PIV: parainfluenza virus 5
RBD: receptor‐binding domain
RFH: refolding hinge
TMD: transmembrane domain

Every year, viral infections impact millions of people worldwide, with both immediate and long‐lasting effects [1]. A wide range of viral diseases impact the global population, involving various pathogens with diverse transmission modes. Examples of well‐known pathogenic viruses include influenza, parainfluenza, and SARS‐CoV‐2 [2, 3].

Viral infections can lead to a wide range of symptoms and health implications by targeting different organs. Respiratory viruses, for instance, lead to respiratory tract infections ranging from common colds to severe diseases [4]. Hepatitis viruses mainly affect the liver, leading to chronic liver disease, cirrhosis, or liver cancer [5]. Neurotropic viruses target the central nervous system, causing conditions such as meningitis, encephalitis, or long‐term neurological complications [6]. Vulnerable populations such as young children, the elderly, pregnant women, and immunocompromised people are more susceptible to severe viral infections [7] and areas with lower health standards, crowded living conditions, and limited vaccination coverage are also more affected by virus outbreaks than more developed areas [8].

To spread, a virus must come into contact with a susceptible host. This can happen through respiratory droplets, direct contact, contaminated food or water, or even vector‐borne transmission. Upon reaching the host, the virus will travel until it encounters the host cell, where it binds to specific receptors on its surface. This initial interaction facilitates viral entry that may occur via endocytosis or fusion with the plasma membrane. Once inside the host cell, the virus's genetic material undergoes replication and transcription to generate new viral components for the assembly of new viral particles. Once assembled, new viral particles accumulate within the host cell before being released through cell lysis or budding from the cell membrane (the latter is not immediately fatal to the host cell) [9, 10, 11].

Viral glycoproteins, which are incorporated into the virion during budding or release from the host, are vital for viral entry and infection [12]. Fusion proteins, a specific subset of these glycoproteins, initiate membrane fusion during viral entry by undergoing conformational changes that can be triggered by external factors such as pH reduction, proteolytic cleavage, or electric fields [13, 14].

With this complex mechanism in mind, it becomes clear that studying viral entry, especially the role of fusion proteins, is fundamental in unraveling new strategies to combat viral infections. A diverse array of experimental methods have been used to study viral entry mechanisms. This is the case with X‐ray crystallography [15, 16], cryo‐electron microscopy [17, 18], and live‐cell imaging methods [19, 20], which provide insight into the molecular interactions between viral glycoproteins and host cell receptors, and elucidating mechanisms of viral neutralization, fusion, and entry.

For instance, through X‐ray crystallography data, Yin *et al*. [15] unveiled that substitutions in the parainfluenza virus 5F protein (PIV F protein) influence its fusogenicity. Specifically, the P22L substitution stabilizes the F protein inducing a hypofusogenic state whereas the S443P substitution destabilizes it, resulting in a hyperfusogenic state. Moreover, the molecular details of the SARS‐CoV‐2 spike glycoprotein interaction with its host receptor, ACE2, were described in structural studies using cryo‐EM and a pH‐dependent switch mechanism of receptor recognition and structural changes was unveiled [16, 17]. Besides structural data, live‐cell imaging has also been used to further the knowledge on the dynamic biological process underlying viral infection, enabling the visualization of the early steps of HIV‐1 infection [19] as well as the influenza virus mechanism of Rab11A transport manipulation [20].

Even though experimental techniques can be used to study the molecular properties of these systems, it is difficult to obtain high‐resolution spatial and temporal information about the molecular details of these conformational transitions. This is where computer simulations come in as a valuable tool, not only providing information on the system's dynamics at atomic or quasi‐atomic resolution, but also enabling the study of the systems under conditions that are difficult to probe experimentally. A diverse array of computational models can be employed to understand the conformational dynamics of biomolecules and their interactions. The choice of molecular simulation methods may vary depending on the desired level of detail and the specific properties being studied. Viral entry mechanisms and related events have been typically explored with all‐atom (AA) and coarse‐grain (CG) Molecular Dynamics (MD) simulation approaches. These methodologies are well‐suited to unravel the intricate details governing viral entry, from the virus life cycle and initial viral recognition to the essential conformational changes required for fusion proteins to become fusion‐competent, all the way to the release of the genome into the host cell [10, 21, 22, 23, 24, 25]. For instance, computational modeling of flavivirus dynamics has demonstrated how viral proteins interact with host membranes, providing insights into structural dynamics that are difficult to observe experimentally [25]. MD simulations have also been pivotal in understanding the conformational changes of the SARS‐CoV‐2 spike protein during viral entry, highlighting the critical role of these simulations in deciphering viral mechanisms [26]. Similarly, the structural dynamics of viral fusion proteins throughout the viral life cycle have been extensively explored using MD simulations [10] or even for the design of antiviral therapeuthics [27].

In this review, we provide an overview of the central role of AA and CG MD simulation methods in deciphering viral entry mechanisms, focusing on the importance of fusion proteins in mediating infection.

## Overview of viral entry

In the initial stages of viral entry, the binding of the viral fusion proteins to host cell receptors, which can be proteins, carbohydrates, or lipids, is a crucial phase in the establishment and progression of the infection. Upon binding, viruses may utilize two main entry routes: endocytic or non‐endocytic pathways. The endocytic route involves the recognition of cell surface receptors, a step that triggers the internalization of the virus into the host cell. This is the preferential mode of viral entry for non‐enveloped viruses; however, several enveloped viruses such as influenza, dengue, and ebola also utilize this mechanism to enter the host cell [28, 29, 30, 31]. The non‐endocytic route enables viruses to directly penetrate the plasma membrane under neutral pH conditions [16, 32, 33, 34]. Enveloped viruses such as parainfluenza utilize this mechanism to enter the host cell, and some, such as SARS‐CoV‐2 or HIV [35, 36], can utilize both the non‐endocytic and endocytic pathways for cellular entry.

**Fig. 1:**
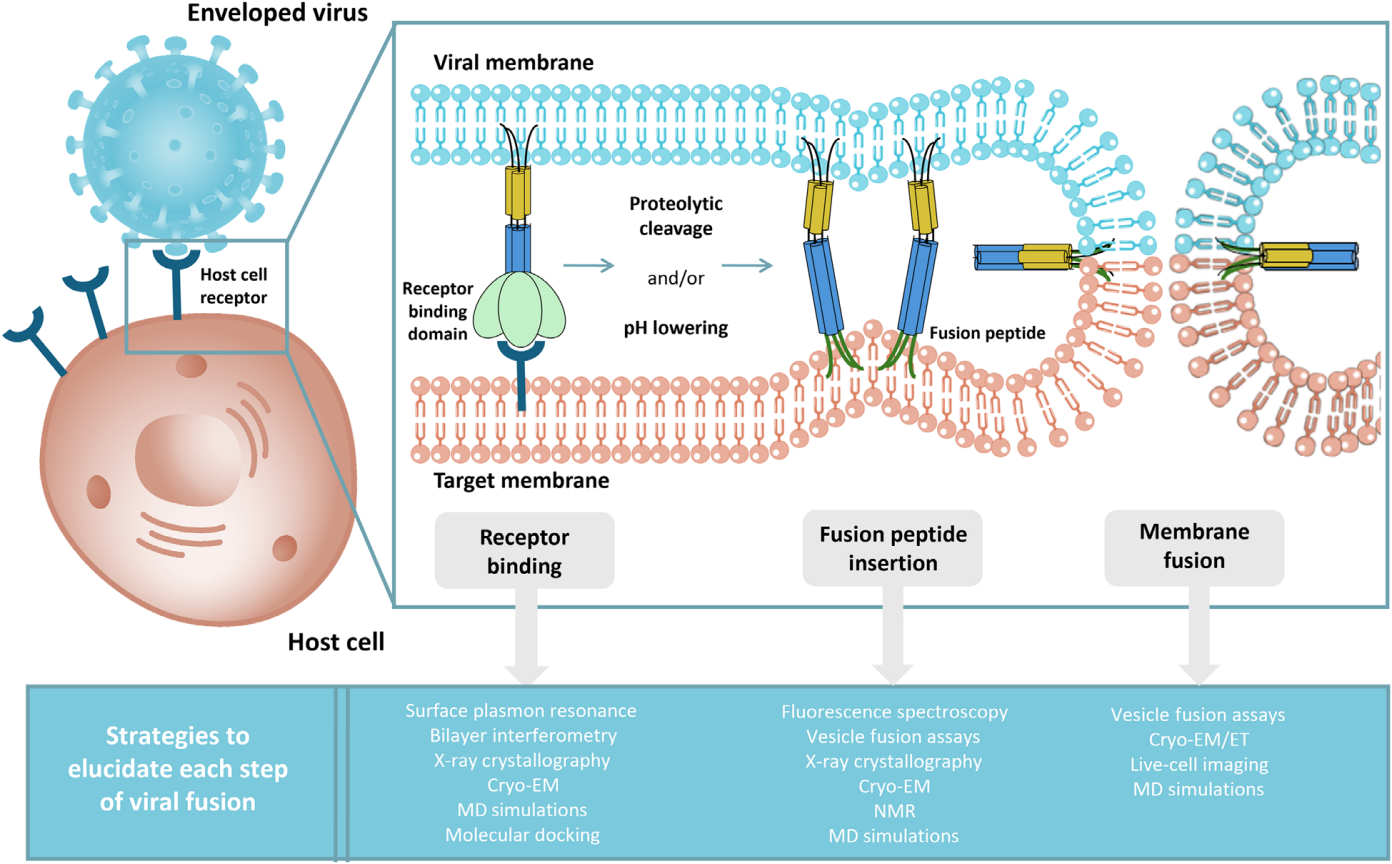
Generic illustration of the successive stages facilitated by viral fusion proteins to enable viral fusion. This depiction highlights the pivotal role of fusion proteins in this process and the conformational alterations they experienced throughout the fusion event. Experimental and computational methodologies can be utilized to describe each phase of this process, which are shown at the bottom of the figure.

An essential process involved in the entry of enveloped viruses, through both endocytic and non‐endocytic routes, is membrane fusion. During viral entry, once the viral and cellular or endocytic membranes approach each other, specific membrane proteins facilitate their fusion. As mentioned earlier, successful entry into host cells requires significant conformational changes in the viral entry proteins or the host cell receptors. These conformational changes, in cases of non‐endocytic entry pathways, can be triggered by various factors, such as the acidic environment within endosomes or proteolytic cleavage. Ultimately, these conformational alterations in the viral fusion proteins of enveloped viruses lead to the fusion of the viral envelope with the host cell membrane (Fig. 1).

In the following sections, we describe the contributions of MD simulations to understanding the intricacies of the viral entry process, conceptually divided into three essential steps—host cell recognition, fusion peptide insertion, and membrane fusion. In all three stages, the viral fusion protein assumes a pivotal role, facilitating the selective binding to the host cell and mediating the fusion of both the host and viral membranes.

## Host cell recognition: finding the target

The first crucial step in viral infection is the recognition process between the virus and the host cell. This initial interaction is mediated by fusion proteins and specific receptors on the surface of target cells. Extensive research has focused on this stage due to its significant impact on viral entry efficiency. The main cell receptors involved in viral entry are proteins or other molecules, such as sialic acid molecules and heparan sulfate proteoglycans, located on the cell surface. Viruses often utilize these receptors to initiate the process of attachment and entry into host cells [11, 37]. The efficiency of the fusion protein‐receptor interaction can be influenced by various factors, including the abundance and distribution of receptors on target cells [38, 39, 40], the conformational changes induced in the fusion protein upon receptor binding [39, 40, 41], and the availability of co‐receptors or other molecules that assist in the fusion process [42].

HIV, for instance, infects human cells through the interaction of its envelope glycoprotein (E) with the host cell receptor CD4 exposed on the cell surface. Upon binding to the CD4, the E protein undergoes conformational changes exposing the V3 loop region able to bind to the co‐receptors CXCR4 and CCR5, initiating the fusion between the viral envelope and the cell membrane [43, 44]. In the case of the influenza virus, the hemagglutinin (HA) protein binds to the sialic acid molecules on the host cell membrane. Although the HA‐sialic acid binding has a low affinity, the high abundance of sialic acid on the host cell surface allows for overall long‐lived interactions. Like the HIV E protein, after binding to its cell receptor, HA suffers profound conformational changes, key for the influenza entry into the target cell [45, 46]. Another example of receptor‐mediated viral entry is the one occurring during SARS‐CoV‐2 infection. The receptor‐binding domain (RBD) within the spike glycoprotein engages the host cell receptor ACE2, which triggers conformational changes in the viral protein [47] and sets off a series of events leading to fusion of the two membranes.

Studies have shown that a strong binding between fusion proteins and cell receptors enhances viral entry efficiency, making it easier for the virus to invade host cells [48, 49]. Given its essential role in infectivity and intercellular fusion, computational tools have been widely utilized to investigate binding affinity. This can be done by analyzing the details of fusion protein‐receptor interactions using molecular docking or molecular dynamics simulations coupled with free energy calculations [50, 51, 52].

Recently, extensive research has been focused on the binding of the SARS‐CoV‐2 fusion protein to its main cell receptor, ACE2. Structural biology studies have identified which residues in the SARS‐CoV‐2 receptor‐binding domain (RBD) are key for ACE2 binding [47, 53], many of which are highly conserved or share similar side‐chain properties with those in the SARS‐CoV RBD [47]. This is supported by computational studies that have shed light on the intricate mechanisms underlying the binding of the RBD to the ACE2 cell receptor [54, 55, 56, 57], as well as potential applications in the pharmaceutical realm [58, 59, 60, 61, 62].

Notably, several works have also contributed to uncovering critical hotspots within the ACE2 receptor, namely K31 and K353 [16, 63, 64]. The same was also done for the RBD and, interestingly, some of the hotspot residues identified to be involved in binding, including K417, F486, Q493, and N501 [64, 65], are mutated in SARS‐CoV‐2 variants. More than identifying the key amino acid residues involved in the RBD‐ACE2 binding, computational approaches have been able to analyze the strength of this interaction and the effect of RBD substitutions in the binding affinity. For example, molecular dynamics simulations have been instrumental in revealing the mechanisms that enhance the binding affinity of the SARS‐CoV‐2 spike protein to the ACE2 receptor across different variants [66]. Additionally, Free Energy Perturbation (FEP) calculations by Sergeeva *et al*. [67] have been used to evaluate the impact of 23 substitutions in the RBD‐ACE2 interface frequently observed in SARS‐CoV‐2 variants. Interestingly, the FEP calculations predicted a stabilization of the RBD‐ACE2 binding in the presence of Q498R and N501Y substitutions detected in Omicron variants, which suggests this may be a mechanistic reason for their selection.

**Fig. 2:**
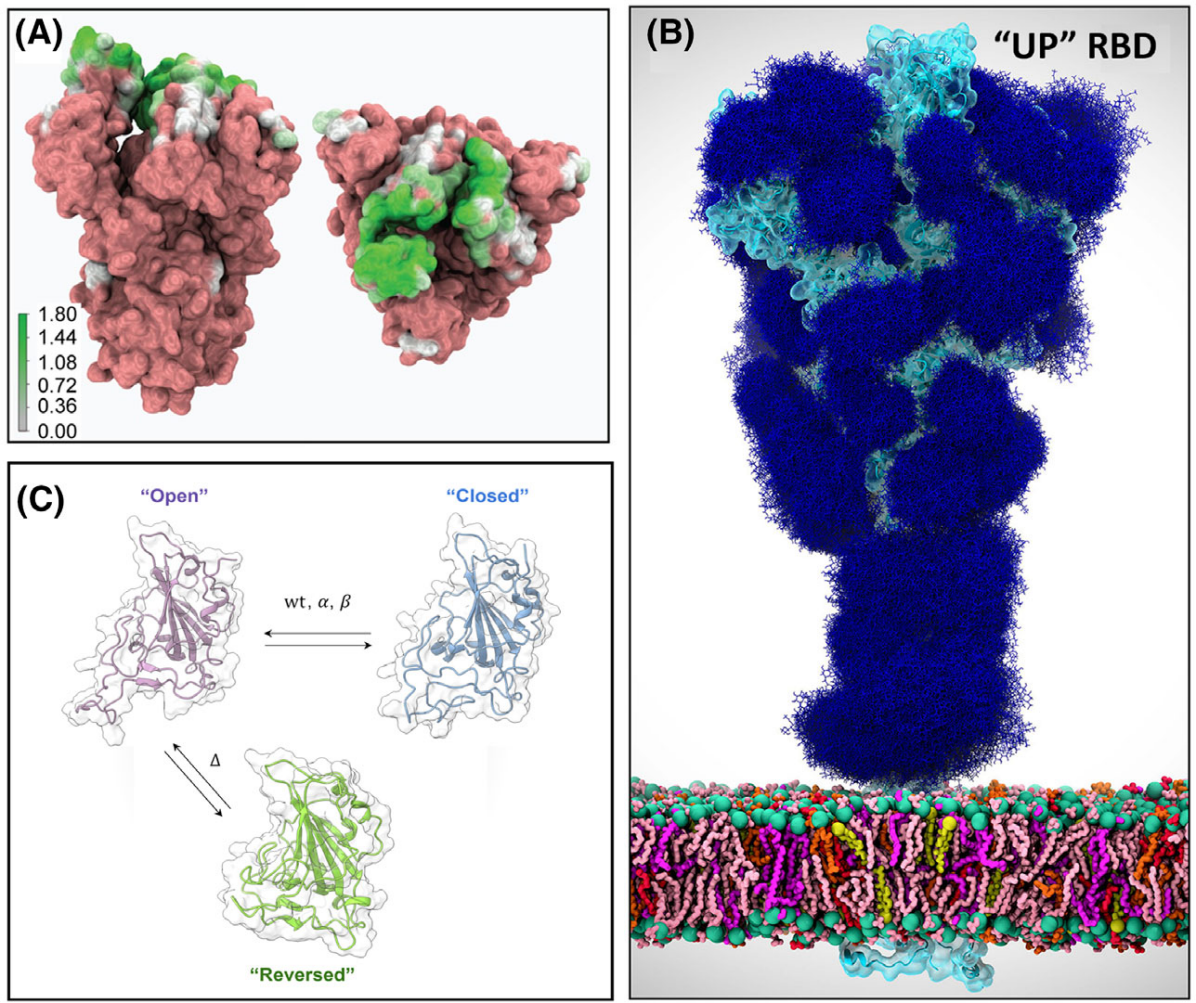
(A) Antibody contact map represented on the spike protein surface, seen from front (left side) and from top (right side); the map was built with VMD [138] and the color gradient is used to represent the log of the frequency of contact with Ab as shown in the scale bar. Reproduced with permission from Ref. [54]. Copyright © 2021 Public Library of Science. (B) Molecular representation of the glycan shield of the SARS‐CoV‐2 S protein in the “up” state. Glycans at several frames (namely, 300 frames, one every 30 ns from one replica) are represented with blue lines, the protein is shown with cartoons and highlighted with a cyan transparent surface. Adapted with permission from Ref. [90]. Copyright © 2020 American Chemical Society. (C) Representation of the “open”, “closed” and “reversed” conformations of the SARS‐CoV‐2 RBD seen by molecular dynamics simulations.

Viral mutations are a common mechanism to evade the immune system; thus, it is crucial to understand the impact of these substitutions not only in the binding to the host cell receptor but also their role in invading the immune system. A comprehensive study combined experimental and computational approaches to identify the effects of single and synergistic amino acid substitutions in promoting immune escape. For example, E484K substitution was found to escape antibody neutralization and this capacity was even increased in the presence of K417N and N501Y substitutions. Moreover, the same study identified S494 as a new amino acid residue of concern, since it shares an identical pattern with E484K, interacting strongly with antibodies but not with the host cell receptor. In the same work, the antibody contact map (shown in Fig. 2A projected onto the spike protein surface) also reveals that the receptor‐binding regions are the most targeted by the immune system [54].

Another key structural feature relevant to target cell binding is the fusion protein's glycosylation [68] (Fig. 2B). Protein glycosylation plays a crucial role in viral pathogenesis [69, 70, 71], as demonstrated by the characteristic N‐glycan coating of the viral fusion proteins [72, 73, 74]. This is the case for the hemagglutinin protein of the influenza A virus. Experimental studies have provided insights into the role of glycosylation in viral infectivity and antigenicity. These have shed light on how glycosylation on hemagglutinin influences receptor binding and virulence. For instance, glycosylation of hemagglutinin seasonal H1N1 influenza A viruses was found to influence virulence and antigenicity, potentially increasing viral fitness and immune evasion [75]. In H3N2 influenza, alterations in the glycosylation pattern of the hemagglutinin's globular head were shown to modulate receptor binding without affecting virulence, suggesting a nuanced role in host adaptation [76]. Similarly, specific glycan modifications on H5N1 hemagglutinin were observed to affect receptor binding and immune response, potentially altering infection dynamics [77]. MD simulations and docking methods can also dissect the structural basis of viral glycan influence on ligand binding by influenza hemagglutinin, offering intricate insights into the intricate interplay between glycosylation and viral infectivity [78, 79].

Glycans on fusion proteins, not only shield the virus from the host immune response but also play a crucial role in the fusion process itself [80, 81, 82, 83, 84]. For instance, the glycosylation of the SARS‐CoV‐2 spike protein has been shown to influence the stability of the protein's interaction with the host membrane and the effectiveness of the fusion process [80, 85, 86]. Computational studies have also elucidated how these glycan structures can either facilitate or hinder the binding of the fusion protein to the host cell, depending on their composition and distribution [87].

In the case of the HIV protein, computational studies have elucidated the influence of N‐linked glycans on the protein dynamics [88] and the importance of glycan shield structures in antibody binding, which contributes to immune evasion [89].

Another example is the SARS‐CoV‐2 fusion protein. It has 22 predicted N‐glycosylation sites per monomer [41, 74], and 20 out of 22 SARS‐CoV‐2N‐linked glycosylation sites are conserved in the SARS‐CoV spike glycoprotein [41]. The surface of the fusion protein is densely glycosylated (Fig. 2B); however, the RBD itself contains only a single glycosylation site, N343 [90]. During the target cell recognition step, dynamic transitions occur in the spike glycoprotein allowing the RBD exposure [91, 92, 93]. Simulation studies have elucidated the conformational transition process of the SARS‐CoV‐2 spike glycoprotein, shifting between its “down” and “up” states [94]. Interestingly, when the RBD is in the “up” state, its glycosylation site becomes relatively distant from the ACE2‐RBD interface. Additionally, a “semi‐intermediate state” has been identified and suggested to be essential for the recognition of the host cell target ACE2.

Several MD simulation works have contributed to the understanding of the role of the spike protein glycans and how they affect protein dynamics. In Casalino *et al*. [90] long AA molecular dynamics simulations reveal the essential role of N‐glycans at sites, N165 and N234, in modulating the conformational dynamics of the S protein RBD. The results of these simulations show that variants with substitutions at these sites display increased instability of RBD in the “up” state and are supported by biolayer interferometry data showing that the deletion of these glycosylation sites reduces binding to ACE2 [90].

The RBD region has also been studied by itself, outside the context of the full spike. Molecular dynamics simulations have explored how naturally occurring mutations in the SARS‐CoV‐2 receptor‐binding domain (RBD) potentially impact target cell recognition and subsequent infection. Mutations in the RBD, even if not directly engaging ACE2, can impact its dynamics. Valério *et al*. [95] have simulated wild‐type RBD and four variants (Alpha, Beta, Delta, Omicron), finding the RBM of the wild‐type RBD transitions between “open” and “closed” conformations (Fig. 2C), with the first favoring ACE2 binding. Alpha and Beta variants increase the open state, likely enhancing ACE2 affinity. Delta and Omicron variants rarely adopt the closed state, with Delta exhibiting an additional “reversed” conformation. This alternate state may aid ACE2 binding and antibody evasion. Overall, mutations in the RBD region affect its dynamics, promoting efficient ACE2 binding and/or antibody escape.

## Fusion peptide insertion: mechanisms and impact

Following target cell recognition, fusion proteins undergo structural changes that lead to the unveiling of the fusion peptide (FP) region. This region is typically located at the N terminus of the fusion protein and plays a pivotal role in facilitating fusion by inserting into and perturbing the host cell membrane [12, 33, 96]. For some fusion proteins, the proteolytic cleavage event is crucial for activation. This allows for the removal of inhibitory regions or structural constraints, allowing the fusion peptide to be exposed and interact with the target cell membrane [33, 96]. This cleavage step is often triggered by the target cell recognition or environmental conditions encountered during viral entry.

**Fig. 3:**
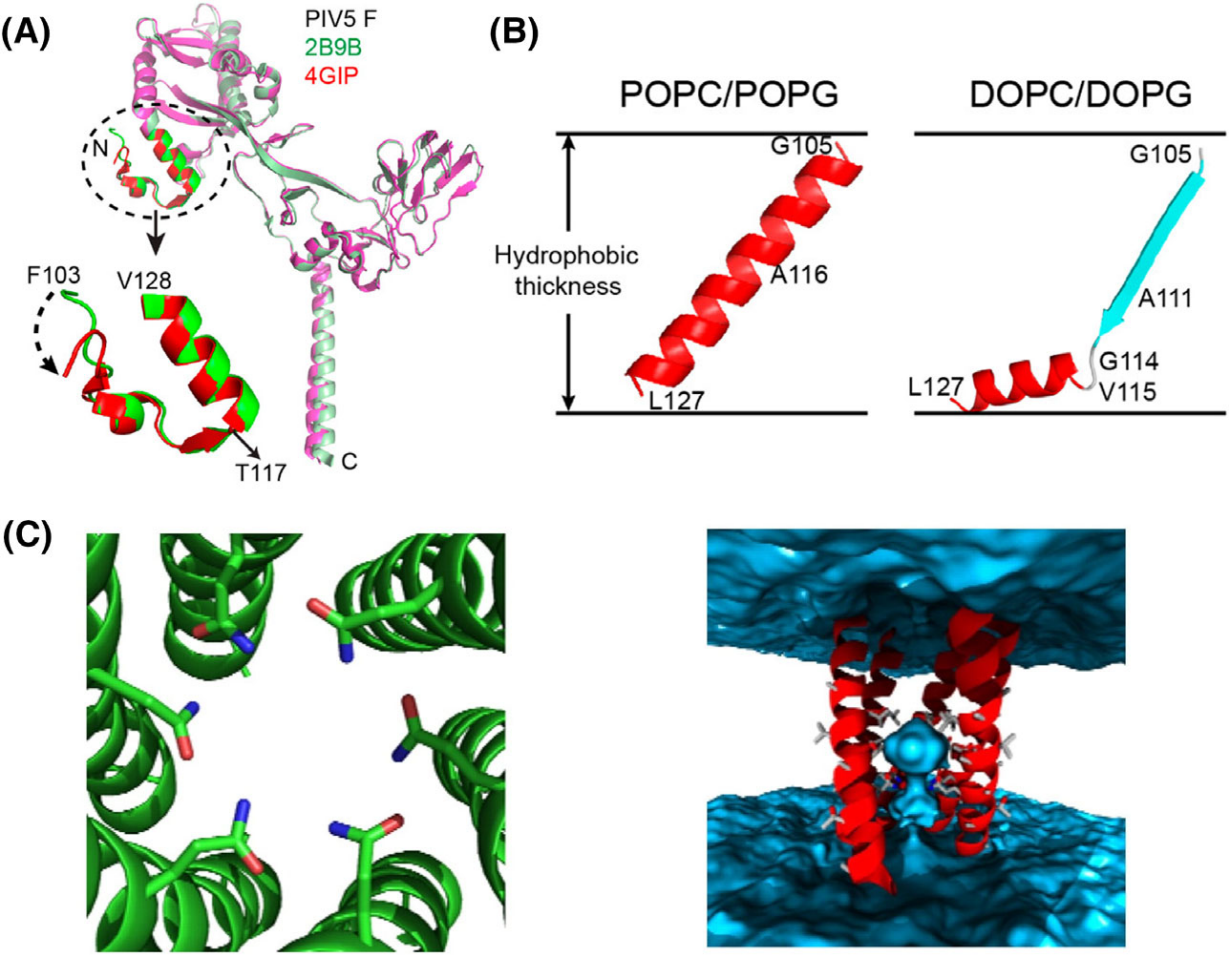
(A) Pre‐fusion crystal structures of the PIV5 F protein in the uncleaved (green) [15] and cleaved (red) [139] states. Figures (A) and (B) were reprinted with permission from [97]. Copyright © 2014 American Chemical Society. (B) PIFP structure determined by SS‐NMR in POPC/POPG and DOPC/DOPG bilayers. The peptide is fully α‐helical in POPC/POPG bilayers, but adopts a mixed strand/helix conformation in DOPC/DOPG bilayers [97]. (C) Snapshot of PIFP hexameric bundle obtained from a short MD simulation (50 ns). The Q120 residues form hydrogen bonds with one another as well as with waters on the interior. Waters are shown in blue, highlighting the penetration of water into the core of the 6HB from the viral side of the membrane. Reprinted with permission from [98]. Copyright © 2011 National Academy of Sciences.

In the pre‐fusion state, fusion peptides are often shielded in pockets within the fusion protein, as is the case for the SARS‐CoV‐2, influenza and parainfluenza fusion peptides. X‐ray crystallography studies of the PIV F protein, revealed that in the pre‐fusion state the FP region is concealed in a cavity of the fusion protein and exhibits a combination of β‐sheet and α‐helical features [15] (Fig. 3A). Upon cleavage and insertion into the target membrane, the PIV fusion peptide (PIFP) extends into a helix. Further details of the conformational dynamics of the PIFP emerged from solid‐state nuclear magnetic resonance investigations of only the peptide. These studies indicate that the PIFP can adopt either α‐helical or β‐strand conformations, depending on the lipid tail composition of the membrane in which it is inserted [97] (Fig. 3B). Simulations have complemented these findings by revealing that in phospholipid micelles, six PIFPs interact in a cooperative and specific manner to assemble into hexamers within membranes, and allow for the penetration of water into its core from the viral side of the membrane (Fig. 3C) [98].

While the sequence of the FPs can differ among various viruses, certain noteworthy characteristics are often conserved. For instance, the fusion peptide commonly contains hydrophobic amino acid residues, allowing it to engage with the lipid bilayer of the host cell membrane. Conservation of hydrophobicity among fusion peptides of the same viral family is also a common feature and highlights its functional importance in fusion [12]. Aromatic residues are commonly found in FP sequences as well. These residues may aid in overcoming the energy cost of peptide partitioning into membranes, especially in non‐N‐terminal fusion peptides. In these cases, the lack of a free N terminus constrains conformational changes in interaction with the membrane surface [99], and the favorable interactions of aromatic side chains with phospholipids may help stabilize the FP sequence insertion into the top bilayer leaflet [100]. Consequently, mutations or alterations to the fusion peptide sequence can have a significant impact on viral infectivity. Assessing the effects of such mutations or alterations can be achieved through infection assays [101] and, complementary, by employing molecular dynamics simulations [102].

Another characteristic shared by fusion peptides is the abundance of alanine and glycine residues [32]. These residues are more abundant in structures with intrinsic conformational flexibility than in more rigid protein domains. This may indicate that sequences of fusion peptides have evolved under selective pressures that extend beyond the constraints imposed by mere hydrophobicity. The insertion depth and angle of fusion peptides inside the membrane can further vary due to factors such as peptide length, membrane lipid composition, especially the presence of anionic phospholipids or cholesterol [97, 103, 104], or pH and ion concentration [105].

**Fig. 4:**
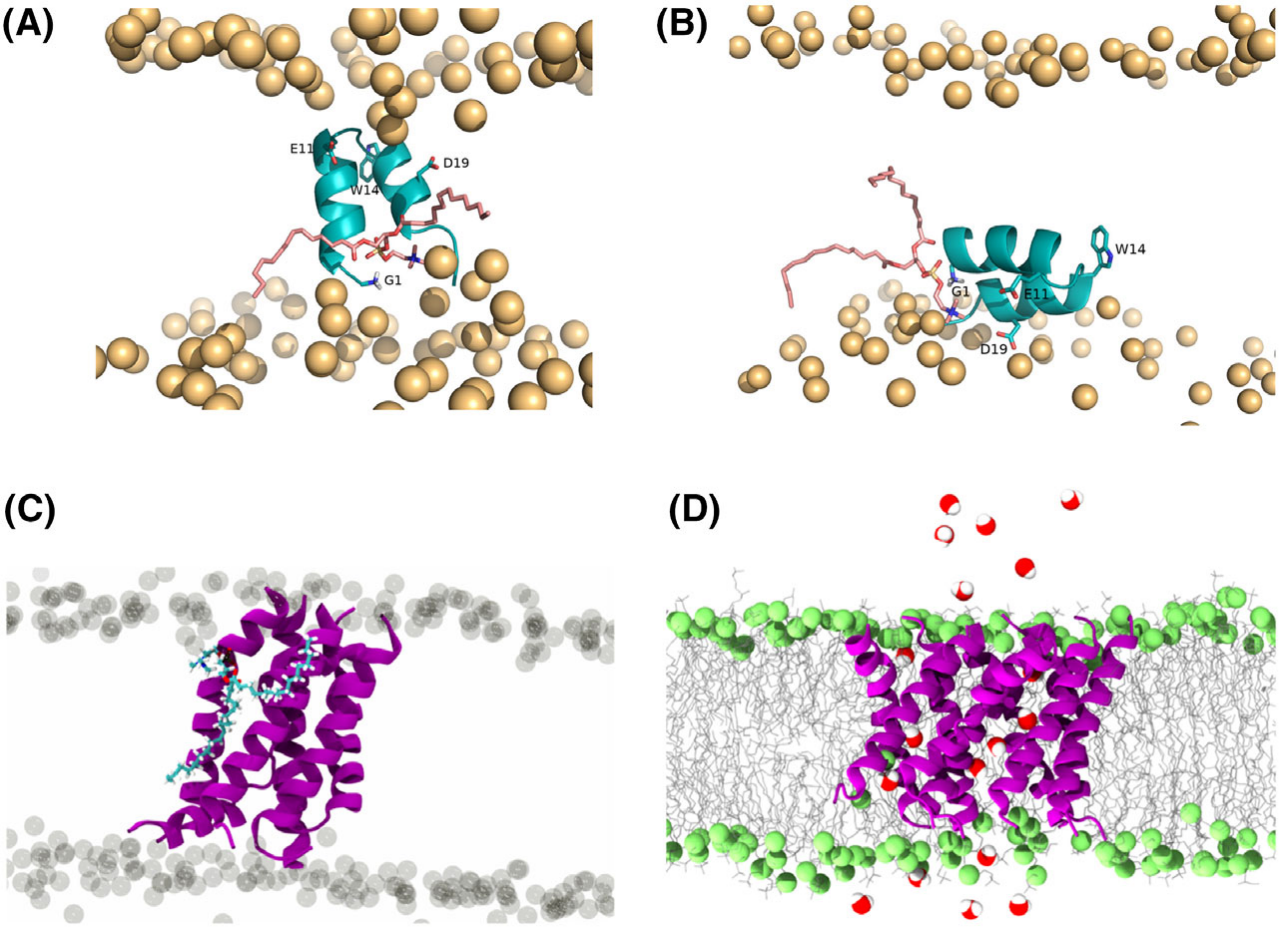
(A) Illustration of a lipid tail protrusion event promoted by interaction of a lipid with the peptide N terminus, observed in the constant‐pH MD simulations performed at pH 5 starting from the vertical conformation. Reprinted with permission from [140]. Copyright © 2021 FEBS Press. (B) Illustration of lipid tail protrusion event promoted by interaction of a lipid with the peptide N terminus, observed in the constant‐pH MD simulations performed at pH 5 starting from the horizontal conformation. (C) Snapshot of the six PIFPs inserted in the membrane, where a lipid is highlighted in blue to evidence the lipid tail protrusion event. (D) Snapshot of the nine PIFPs inserted in the membrane, the water crossing the pore‐like structure formed by the peptides are highlighted. Figures (C) and (D) were reprinted with permission from [119]. Copyright © 2022 American Chemical Society.

The effect of peptide length on the FPs membrane insertion is particularly evident in the case of the influenza fusion peptide (IFP), a widely studied fusion peptide that serves as a benchmark for understanding other N‐terminal fusion peptides. NMR studies of the 20‐residue long IFP in a lipid environment have provided insights into its structural behavior. Under fusogenic conditions (pH 5), the peptide adopts a helical inverted V structure when placed in detergent micelles. However, at pH 7.4, it takes on a less structured conformation in its C‐terminal region [106]. In contrast, recent research has shown that an IFP with only 3 residues more (23 residues instead of 20), forms a tightly wound helical hairpin structure at both pH 4 and 7.4 [107]. This highlights how the addition of just three amino acid residues—which are conserved within the fusion peptide family [108]—can significantly influence the fusion peptide's structure and its interaction with membranes. Molecular dynamics simulations have played a pivotal role in exploring the structural characteristics and orientation of IFP within lipid membranes. In many studies, the fusion peptide is initially positioned at the interface between lipid head groups and lipid tails, following NMR observations. In these simulations, the peptide generally remains in this region throughout the simulation duration [109, 110, 111, 112], something that likely occurs due to the limited time simulated for these systems. Therefore, this approach may not fully capture the peptide's dynamic behavior because it restricts its ability to explore alternative states due to substantial energy barriers associated with such transitions. To overcome this limitation, Victor *et al*. [113] employed a self‐assembly approach. In this approach, lipids and water molecules start the simulation from random positions and spontaneously assemble—around the peptide, if its membrane insertion is energetically favorable. In most replicates, the peptide adopted a membrane‐spanning helix‐turn‐helix configuration (Fig. 4A), although one remained at the interface between lipid headgroups and tails (see Fig. 4B). Replica‐exchange molecular dynamics simulations further supported the hypothesis that the influenza FP can exist in two configurations, with the membrane‐spanning form being the lowest energy state for the 23‐residue peptide with a charged N terminus [114, 115].

## Viral membrane fusion: role of lipid tail protrusion

The membrane‐spanning conformation of the IFP, besides being the lowest energy state for the 23‐residue peptide with a charged N terminus, is also the conformation that causes greater membrane disruption and lipid tail protrusion [102, 116] (Fig. 4, lipid in pink). Induction of lipid tail protrusion has not only been seen through molecular dynamics, but recent NMR studies have also shown that the IFP can cause lipid acyl chain protrusion, a key event in the fusion process [117]. These events are not a specific mechanism of IFP and are thought to be induced by viral fusion peptides during membrane fusion to lower the energy barrier for fusing the two juxtaposed membranes. Lipid tail protrusion events are caused by FP insertion in the lipid bilayer, disrupting the native bilayer organization and leading the lipid tails to protrude outward from the membrane surface. Lipid tail protrusion was first described in MD simulations studies and predicted to drive lipid mixing and the fusion stalk formation [118]. Due to the quick and transient character of lipid tail protrusion, experimental detection is difficult and usually only the associated phenomena of lipid mixing or fusion are observed. For instance, MD simulations of the PIFP revealed its ability to induce lipid tail protrusion (Fig. 4C) in a concentration‐dependent manner by the formation of oligomeric structures within the membrane that destabilized the membrane organization and allow for the passage of water molecules (Fig. 4D). In the same work, experimental biophysics methods corroborated the *in silico* insights by showing that the PIFP also promoted lipid mixing in a similar manner [119].

Beyond lipid tail protrusion, viral fusion proteins interact with target membranes in complex ways that affect membrane dynamics and stability. For instance, viral fusion proteins often exploit the host cell membrane's physical properties to promote fusion [120, 121, 122, 123]. Unsaturated lipids or cholesterol molecules, for example, have been shown to accelerate the formation of oligomeric β‐sheet structures or alter membrane physical properties, respectively, thereby enhancing GP41 FP activity [120, 121].

Viral fusion peptides can also influence membrane curvature, modify the area per lipid, create local instabilities, and even lead to the formation of non‐lamellar phases that facilitate membrane fusion [124, 125, 126, 127]. For instance, studies have shown that viral fusion proteins can sculpt host membranes through the insertion of charged and polar groups, which destabilizes the membrane and facilitates fusion [128]. Viral capsomers have also been demonstrated to induce significant deformations in the host membrane, potentially aiding in the entry of the viral genome into the cell [129]. Recently, Rice *et al*. [130] demonstrated that the planar aggregation of the IFP significantly alters membrane structure and hydration, promoting membrane fusion.

Besides FPs‐lipid interactions, other important ones may contribute to membrane fusion. For instance, some viral FPs require intrapeptide interactions or ion binding to enhance their fusogenic activity. In the case of the SARS‐CoV‐2 FP, CG MD simulations showed how the disulfide bond formed between the Cys840 and Cys851 stabilizes its C‐terminal helical structure which, in consequence, enhances the binding between the FP and the lipid bilayer [131]. Furthermore, SARS‐CoV‐2 FP propensity to form a helical structure as well as its fusogenicity is increased by the binding to calcium ions, which has been shown by MD simulations to depend on the pairs Glu819/Asp820 and Asp830/Asp839 [132].

Studying the viral FPs isolated both *in silico* and *in vitro* has proven to yield helpful insights into understanding the mechanisms of membrane fusion induced by these peptides. However, viral FPs exist in the context of a viral fusion protein and, thus some features may not be assessed by studying the FPs isolated. MD simulations are valuable tools to address this issue providing atomic details of the membrane fusion process even with a full‐length viral fusion protein.

**Fig. 5:**
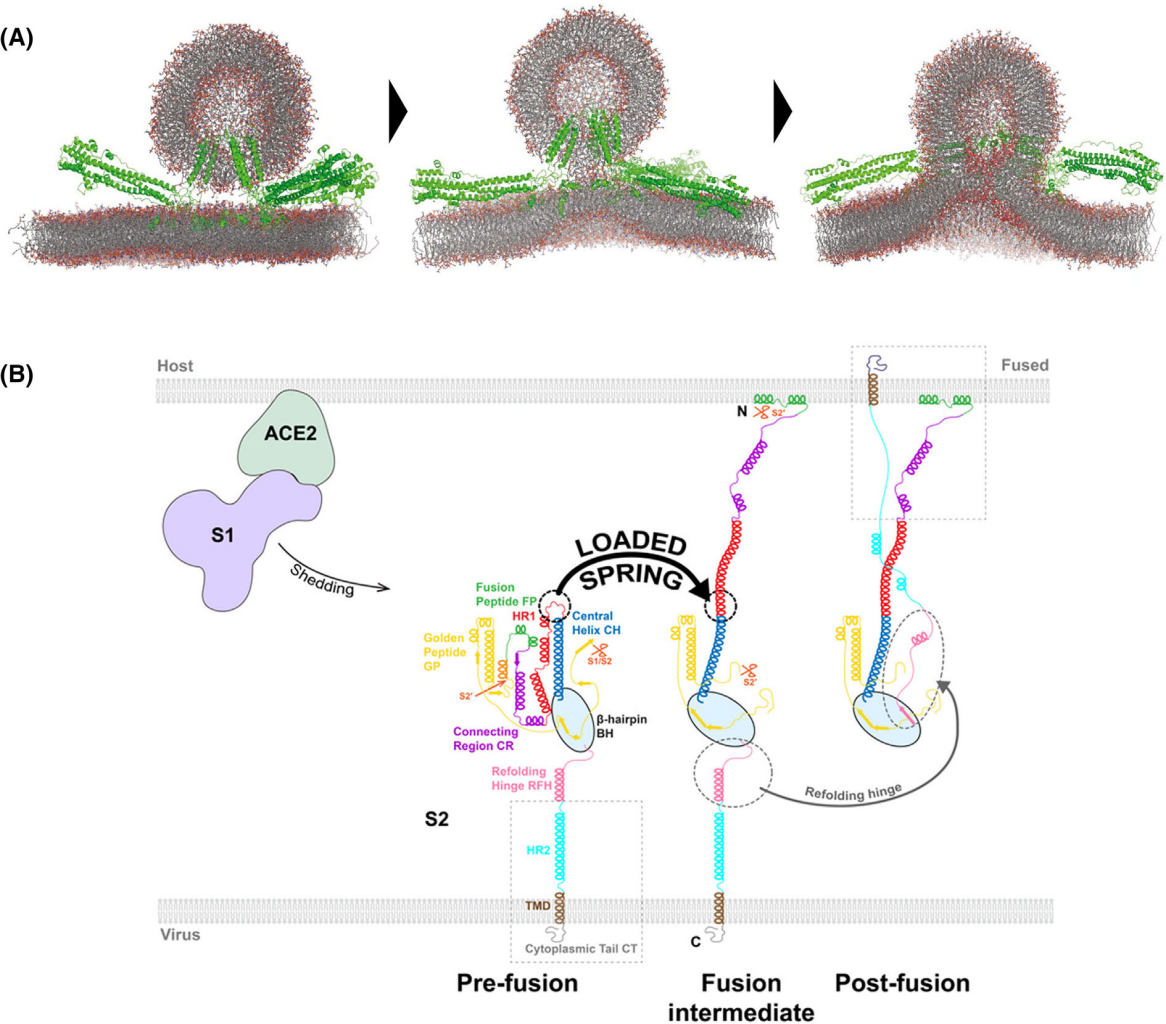
(A) Atomic‐resolution simulations of a small proteoliposome docked to a planar bilayer by three full‐length hemagglutinin trimers resulted in fusion stalk formation, and finally a nascent fusion pore (from left to right). Adapted with permission from [133]. Copyright © 2020 Proceedings of the National Academy of Sciences. (B) Model of the SARS‐CoV‐2 spike protein pre‐ to post‐fusion transition. Following the dissociation of S2 from S1, the unstructured pre‐fusion HR1 loops become helical (loaded spring release), giving the HR1‐CH backbone that thrusts the FPs toward the host cell membrane. The FI subsequently refolds into the post‐fusion structure. Adapted with permission from [135]. Copyright © 2023 American Chemical Society.

Pabis *et al*. [133] have used a multiresolution approach consisting in AA and CG simulations to study the mechanism of fusion between a planar bilayer and a influenza HA proteoliposome. These simulations containing a proteoliposome with three full‐length HA trimers and a planar bilayer began from a docked state after HA ectodomain refolding, with the FPs already inserted in the target bilayer. HA trimers start by increasing the lipid tail protrusion, leading to the stalk formation. Then, each distal leaflet is engaged by the HA inducing a widening of the stalk along with an increase in membrane curvature. Ultimately, membrane fusion between the planar bilayer and the proteoliposome is achieved (Fig. 5A). Importantly, these results highlight the intramembrane role of the IFP in driving the fusion process [133]. Moreover, the full‐length HA model allowed to understand the mutational sensitivity of the IFP, previously pointed out by mutational and biophysical data [112, 134].

Additionally, in the work of Pabis *et al*. [133], part of the HA conformational changes that lead to the stalk and fusion pore formation were observed. The dynamic structure of viral fusion proteins is a key feature to overcome energetic barriers involved in membrane fusion. Thus, understanding the mechanisms of conformational changes of viral proteins provides fundamental insights of the viral entry process. To achieve this level of detail, Su *et al*. [135] have built a full‐length model of the fusion intermediate of SARS‐CoV‐2 spike glycoprotein by extrapolation based on pre‐ and post‐fusion structures already solved. This model incorporated the S2 domains: cytoplasmic tail (CT), transmembrane domain (TMD), heptad repeat 2 (HR2), refolding hinge (RFH), beta‐hairpin (BH), golden peptide (GP), S2’ cleavage site, fusion peptide (FP), connecting region (CR), heptad repeat 1 (HR1) and the central helix (CH). The FP, CR, and RFH structure was retrieved from the spike glycoprotein pre‐fusion conformation, while GP, HR1, CH, and BH were obtained from post‐fusion conformation structures. The HR2 structure used belongs to the SARS‐CoV, since it is identical to SARS‐CoV‐2 HR2. Both TMD and CT structures were predicted using QUARK [136], due to the lack of solved structures for these regions. The model was designed to capture the structural dynamics and flexibility of the fusion intermediate. In fact, CG MD simulations showed drastic bending and extensional fluctuations as the result of the three RFH, suggesting a “loaded spring” mechanism enabling the pre‐ to post‐fusion transition (Fig. 5B). Moreover, this movement significantly boosts the volume searched by the fusion intermediate allowing it to target and engage the host membrane in approximately 2 ms. This insight showcases the powerful and indispensable role of MD simulations in providing mechanistic detailed information on processes occurring at a short timescale, often difficult to follow in experimental conditions.

## Overall outlook

Viral entry is a critical step in the viral infection cycle and is a complex process involving interactions between the viral fusion protein and host cell receptors, major fusion protein conformational changes and membrane fusion. Computational studies, particularly MD simulations have been crucial in providing molecular details on the viral fusion protein‐receptor interactions, on the fusion peptide insertion and its effect on the lipid bilayer and even on the fusion protein structural changes that occur during fusion. These molecular mechanistic details provide fundamental knowledge of viral entry for a variety of viruses that can be leveraged for the fighting of viral infections.

This has been done by several groups worldwide, among them the David Baker group, which has made significant breakthroughs by leveraging the knowledge on the SARS‐CoV‐2 RBD interaction with the ACE2 host cell receptor. Baker's lab used computational protein design methods to design viral inhibitors targeting the SARS‐CoV‐2 RBD [58]. Two major approaches were performed: in the first one, the ACE2 RBD‐binding segment was incorporated into small scaffold proteins and; in the second one, proteins were designed from scratch to bind to the RBM of the RBD. Both strategies resulted in highly effective inhibitors. In particular, the *de novo* protein design generated viral inhibitors such as LCB1 and LCB3 with an inhibiting potency in the pM range, outperforming existing neutralizing antibodies [58]. Furthermore, to combat the emerging SARS‐CoV‐2 variants of concern, including Omicron and Delta, Baker's team designed multivalent minibinder proteins enhancing viral neutralization. Notably, one of the multivalent binders, the TRI2‐2, demonstrated potency across all variants tested, highlighting the versatility and effectiveness of the new generation of designed inhibitors [137]. Overall, this work is a successful example of how the fundamental knowledge on viral entry can be utilized to respond to current and emerging viral threats.

In conclusion, computational simulations offer powerful tools to enhance our understanding of viral entry, unraveling the molecular mechanisms of viral fusion proteins and host cell interactions. The future insights will provide promising new targets for the development of a novel generation of viral inhibitors crucial for combating viral infections and mitigating their global health impact.

## Conflict of interest

The authors declare that they have no known competing financial interests or personal relationships that could have appeared to influence the work reported in this paper.

## Author contributions

MV, CCB, MNM, CMS, and DL defined the scope of this review, the topics that would be covered and the perspective that would be adopted; MV and CCB reviewed the literature; MV, CCB, MNM, CMS and DL wrote and reviewed the manuscript.

## References

[1] Zhang S‐Y , Jouanguy E , Sancho‐Shimizu V , von Bernuth H , Yang K , Abel L , Picard C , Puel A and Casanova J‐L (2007) Human toll‐like receptor‐dependent induction of interferons in protective immunity to viruses. Immunol Rev 220, 225–236. 17979850 10.1111/j.1600-065X.2007.00564.x PMC7165931

[2] Morens DM and Al E (2020) The COVID‐19 pandemic: what can we learn from it? J Clin Invest 130, 2140–2145.

[3] Simons E , Ferrari M , Fricks J , Wannemuehler K , Anand A , Burton A and Strebel P (2012) Assessment of the 2010 global measles mortality reduction goal: results from a model of surveillance data. Lancet 379, 2173–2178. 22534001 10.1016/S0140-6736(12)60522-4

[4] Fields BN , Knipe DM , Howley PM and Griffin DE (2007) Fields' Virology. Lippincott Williams & Wilkins.

[5] Patel MM and Al E (2008) Global epidemiology of hepatitis a virus infection: new estimates of age‐specific antibody to HAV seroprevalence. Hepatology 48, 1333–1341. 23172780 10.1002/hep.26141

[6] Solomon T , Dung NM , Vaughn DW , Kneen R , Thao LT , Raengsakulrach B , Loan HT , Day NP , Farrar J , Myint KS *et al*. (2000) Neurological manifestations of dengue infection. Lancet 355, 1053–1059. 10744091 10.1016/S0140-6736(00)02036-5

[7] Clark A , Jit M , Warren‐Gash C , Guthrie B , Wang HHX , Mercer SW , Sanderson C , McKee M , Troeger C , Ong KL *et al*. (2020) Global, regional, and national estimates of the population at increased risk of severe COVID‐19 due to underlying health conditions in 2020: a modelling study. Lancet Glob Health 8, e1003–e1017. 32553130 10.1016/S2214-109X(20)30264-3 PMC7295519

[8] UNICEF (2019) Progress on household drinking water, sanitation, and hygiene 2000–2017: Special focus on inequalities. UNICEF, New York, NY, USA.

[9] Dimitrov DS (2004) Virus entry: molecular mechanisms and biomedical applications. Nat Rev Microbiol 2, 109–122. 15043007 10.1038/nrmicro817 PMC7097642

[10] Jones JE , Le Sage V and Lakdawala SS (2021) Viral and host heterogeneity and their effects on the viral life cycle. Nat Rev Microbiol 19, 272–282. 33024309 10.1038/s41579-020-00449-9 PMC7537587

[11] Maginnis MS (2018) Virus – receptor interactions: the key to cellular invasion. J Mol Biol 430, 2590–2611. 29924965 10.1016/j.jmb.2018.06.024 PMC6083867

[12] White JM and Whittaker GR (2016) Fusion of enveloped viruses in endosomes. Traffic 17, 593–614. 26935856 10.1111/tra.12389 PMC4866878

[13] Benton DJ , Gamblin SJ , Rosenthal PB and Skehel JJ (2020) Structural transitions in influenza haemagglutinin at membrane fusion pH. Nature 583, 150–153. 32461688 10.1038/s41586-020-2333-6 PMC7116728

[14] Lipskij A , Arbeitman C , Rojas P , Ojeda‐May P and Garcia ME (2023) Dramatic differences between the structural susceptibility of the S1 pre‐ and S2 Postfusion states of the SARS‐CoV‐2 spike protein to external electric Fields revealed by molecular dynamics simulations. Viruses 15, 2405. 38140646 10.3390/v15122405 PMC10748067

[15] Yin H‐S , Wen X , Paterson RG , Lamb RA and Jardetzky TS (2006) Structure of the parainfluenza virus 5 F protein in its metastable, prefusion conformation. Nature 439, 38–44. 16397490 10.1038/nature04322 PMC7095149

[16] Shang J , Ye G , Shi K , Wan Y , Luo C , Aihara H , Geng Q , Auerbach A and Li F (2020) Structural basis of receptor recognition by SARS‐CoV‐2. Nature 581, 221–224. 32225175 10.1038/s41586-020-2179-y PMC7328981

[17] Zhou T , Tsybovsky Y , Gorman J , Rapp M , Cerutti G , Chuang GY , Katsamba PS , Sampson JM , Schön A , Bimela J *et al*. (2020) Cryo‐EM structures of SARS‐CoV‐2 spike without and with ACE2 reveal a pH‐dependent switch to mediate endosomal positioning of receptor‐binding domains. Cell Host Microbe 28, 867–879.e5. 33271067 10.1016/j.chom.2020.11.004 PMC7670890

[18] Dam K‐MA , Fan C , Yang Z and Bjorkman PJ (2024) Publisher correction: intermediate conformations of CD4‐bound HIV‐1 Env heterotrimers. Nature 626, E7. 38243106 10.1038/s41586-024-07082-z PMC10849941

[19] Francis AC and Melikyan GB (2018) Live‐cell imaging of early steps of single HIV‐1 infection. Viruses 10, 275. 29783762 10.3390/v10050275 PMC5977268

[20] Bhagwat AR , Le Sage V , Nturibi E , Kulej K , Jones J , Guo M , Tae Kim E , Garcia BA , Weitzman MD , Shroff H *et al*. (2020) Quantitative live cell imaging reveals influenza virus manipulation of Rab11A transport through reduced dynein association. Nat Commun 11, 23. 31911620 10.1038/s41467-019-13838-3 PMC6946661

[21] Yokoyama M , Fujisaki S , Shirakura M , Watanabe S , Odagiri T , Ito K and Sato H (2017) Molecular dynamics simulation of the influenza a(H3N2) hemagglutinin trimer reveals the structural basis for adaptive evolution of the recent epidemic clade 3C.2a. Front Microbiol 8, 584. 28443077 10.3389/fmicb.2017.00584 PMC5385362

[22] Durrant JD , Kochanek SE , Casalino L , Ieong PU , Dommer AC and Amaro RE (2020) Mesoscale all‐atom influenza virus simulations suggest new substrate binding mechanism. ACS Cent Sci 6, 189–196. 32123736 10.1021/acscentsci.9b01071 PMC7048371

[23] Fujimoto K , Yamaguchi Y , Urano R , Shinoda W , Ishikawa T , Omagari K , Tanaka Y , Nakagawa A and Okazaki S (2021) All‐atom molecular dynamics study of hepatitis B virus containing pregenome RNA in solution. J Chem Phys 155, 145101. 34654297 10.1063/5.0065765

[24] Casalino L , Seitz C , Lederhofer J , Tsybovsky Y , Wilson IA , Kanekiyo M and Amaro RE (2022) Breathing and tilting: mesoscale simulations illuminate influenza glycoprotein vulnerabilities. ACS Cent Sci 8, 1646–1663. 36589893 10.1021/acscentsci.2c00981 PMC9801513

[25] Huber RG , Marzinek JK , Boon PLS , Yue W and Bond PJ (2021) Computational modelling of flavivirus dynamics: the ins and outs. Methods 185, 28–38. 32526282 10.1016/j.ymeth.2020.06.004 PMC7278654

[26] Abduljalil JM , Elghareib AM , Samir A , Ezat AA and Elfiky AA (2023) How helpful were molecular dynamics simulations in shaping our understanding of SARS‐CoV‐2 spike protein dynamics? Int J Biol Macromol 242, 125153. 37268078 10.1016/j.ijbiomac.2023.125153 PMC10232722

[27] Dzimianski JV , Han J , Sautto GA , O'Rourke SM , Cruz JM , Pierce SR , Ecker JW , Carlock MA , Nagashima KA , Mousa JJ *et al*. (2023) Structural insights into the broad protection against H1 influenza viruses by a computationally optimized hemagglutinin vaccine. Communications Biology 6, 1–13. 37185989 10.1038/s42003-023-04793-3 PMC10126545

[28] Mercer J and Helenius A (2010) Virus entry by endocytosis. Annu Rev Biochem 79, 803–833. 20196649 10.1146/annurev-biochem-060208-104626

[29] Sieczkarski SB and Whittaker GR (2002) Influenza virus can enter and infect cells in the absence of clathrin‐mediated endocytosis. J Virol 76, 10455–10464. 12239322 10.1128/JVI.76.20.10455-10464.2002 PMC136567

[30] Acosta EG and Bartenschlager R (2009) The multifaceted role of clathrin‐mediated endocytosis during viral infection. Trends Microbiol 17, 590–597.

[31] Doherty GJ and McMahon HT (2009) Mechanisms of endocytosis. Annu Rev Biochem 78, 857–902. 19317650 10.1146/annurev.biochem.78.081307.110540

[32] White JM (1992) Membrane fusion. Science 258, 917–924. 1439803 10.1126/science.1439803

[33] Harrison SC (2008) Viral membrane fusion. Nat Struct Mol Biol 15, 690–698. 18596815 10.1038/nsmb.1456 PMC2517140

[34] Belouzard S , Chu VC and Whittaker GR (2009) Entry route of the enveloped virus hepatitis C virus depends on clathrin‐mediated endocytosis. J Virol 83, 398–411.

[35] Yuan M , Huang D , Lee C‐CD , Wu NC , Jackson AM , Zhu X , Liu H , Peng L , Van Gils MJ , Sanders RW *et al*. (2021) Structural and functional ramifications of antigenic drift in recent SARS‐CoV‐2 variants. Science 373, 818–823. 34016740 10.1126/science.abh1139 PMC8284396

[36] Miyauchi K , Kim Y , Latinovic O , Morozov V and Melikyan GB (2009) HIV enters cells via endocytosis and dynamin‐dependent fusion with endosomes. Cell 137, 433–444. 19410541 10.1016/j.cell.2009.02.046 PMC2696170

[37] Grove J and Marsh M (2011) The cell biology of receptor‐mediated virus entry. J Cell Biol 195, 1071–1082. 22123832 10.1083/jcb.201108131 PMC3246895

[38] Ivanovic T , Choi JL , Whelan SP , van Oijen AM and Harrison SC (2013) Influenza‐virus membrane fusion by cooperative fold‐back of stochastically induced hemagglutinin intermediates. eLife 2, e00333. 23550179 10.7554/eLife.00333 PMC3578201

[39] Cai Y , Zhang J , Xiao T , Peng H , Sterling SM , Walsh RM Jr , Rawson S , Rits‐Volloch S and Chen B (2020) Distinct conformational states of SARS‐CoV‐2 spike protein. Science 369, 1586–1592. 32694201 10.1126/science.abd4251 PMC7464562

[40] Chao LH , Klein DE , Schmidt AG , Peña JM and Harrison SC (2014) Sequential conformational rearrangements in flavivirus membrane fusion. eLife 3, e04389. 25479384 10.7554/eLife.04389 PMC4293572

[41] Walls AC , Park YJ , Tortorici MA , Wall A , McGuire AT and Veesler D (2020) Structure, function, and antigenicity of the SARS‐CoV‐2 spike glycoprotein. Cell 181, 281–292.e6. 32155444 10.1016/j.cell.2020.02.058 PMC7102599

[42] White JM , Delos SE , Brecher M and Schornberg K (2008) Structures and mechanisms of viral membrane fusion proteins: multiple variations on a common theme. Crit Rev Biochem Mol Biol 43, 189–219. 18568847 10.1080/10409230802058320 PMC2649671

[43] Wyatt R and Sodroski J (1998) The HIV‐1 envelope glycoproteins: fusogens, antigens, and immunogens. Science 280, 1884–1888. 9632381 10.1126/science.280.5371.1884

[44] Kwong PD (2000) Structures of HIV‐1 gp120 envelope glycoproteins from laboratory‐adapted and primary isolates. Structure 8, 1329–1339. 11188697 10.1016/s0969-2126(00)00547-5

[45] Skehel JJ and Wiley DC (2000) Receptor binding and membrane fusion in virus entry: the influenza hemagglutinin. Annu Rev Biochem 69, 531–569. 10966468 10.1146/annurev.biochem.69.1.531

[46] Gamblin SJ and Skehel JJ (2010) Influenza hemagglutinin and neuraminidase membrane glycoproteins. J Biol Chem 285, 28403–28409. 20538598 10.1074/jbc.R110.129809 PMC2937864

[47] Lan J , Ge J , Yu J , Shan S , Zhou H , Fan S , Zhang Q , Shi X , Wang Q , Zhang L *et al*. (2020) Structure of the SARS‐CoV‐2 spike receptor‐binding domain bound to the ACE2 receptor. Nature 581, 215–220. 32225176 10.1038/s41586-020-2180-5

[48] Barton MI , MacGowan SA , Kutuzov MA , Dushek O , Barton GJ and van der Merwe PA (2021) Effects of common mutations in the SARS‐CoV‐2 spike RBD and its ligand, the human ACE2 receptor on binding affinity and kinetics. eLife 10, e70658. 34435953 10.7554/eLife.70658 PMC8480977

[49] Hasegawa K , Hu C , Nakamura T , Marks JD , Russell SJ and Peng K‐W (2007) Affinity thresholds for membrane fusion triggering by viral glycoproteins. J Virol 81, 13149–13157. 17804513 10.1128/JVI.01415-07 PMC2169077

[50] Siebenmorgen T and Zacharias M (2020) Computational prediction of protein–protein binding affinities. Wiley Interdiscip Rev Comput Mol Sci 10, 1448.

[51] Kumar V , Singh J , Hasnain SE and Sundar D (2021) Possible link between higher transmissibility of alpha, kappa and delta variants of SARS‐CoV‐2 and increased structural stability of its spike protein and hACE2 affinity. Int J Mol Sci 22, 9131. 34502041 10.3390/ijms22179131 PMC8431609

[52] Govind Kumar V , Polasa A , Agrawal S , Kumar TKS and Moradi M (2023) Binding affinity estimation from restrained umbrella sampling simulations. Nat Comput Sci 3, 59–70. 38177953 10.1038/s43588-022-00389-9 PMC10766565

[53] Yan R , Zhang Y , Li Y , Xia L , Guo Y and Zhou Q (2020) Structural basis for the recognition of SARS‐CoV‐2 by full‐length human ACE2. Science 367, 1444–1448. 32132184 10.1126/science.abb2762 PMC7164635

[54] Alenquer M , Ferreira F , Lousa D , Valério M , Medina‐Lopes M , Bergman ML , Gonçalves J , Demengeot J , Leite RB , Lilue J *et al*. (2021) Signatures in SARS‐CoV‐2 spike protein conferring escape to neutralizing antibodies. PLoS Pathog 17, e1009772. 34352039 10.1371/journal.ppat.1009772 PMC8341613

[55] Lupala CS , Li X , Lei J , Chen H , Qi J , Liu H and Su X‐D (2021) Computational simulations reveal the binding dynamics between human ACE2 and the receptor binding domain of SARS‐CoV‐2 spike protein. Quantitative Biology 9, 61.

[56] Yan FF and Gao F (2021) Comparison of the binding characteristics of SARS‐CoV and SARS‐CoV‐2 RBDs to ACE2 at different temperatures by MD simulations. Brief Bioinform 22, 1122–1136. 33611368 10.1093/bib/bbab044 PMC7929385

[57] Xu C , Wang Y , Liu C , Zhang C , Han W , Hong X , Wang Y , Hong Q , Wang S , Zhao Q *et al*. (2021) Conformational dynamics of SARS‐CoV‐2 trimeric spike glycoprotein in complex with receptor ACE2 revealed by cryo‐EM. Science Advances 7, eabe5575. 33277323 10.1126/sciadv.abe5575 PMC7775788

[58] Cao L , Goreshnik I , Coventry B , Case JB , Miller L , Kozodoy L , Chen RE , Carter L , Walls AC , Park YJ *et al*. (2020) De novo design of picomolar SARS‐CoV‐2 miniprotein inhibitors. Science 370, 426–431. 32907861 10.1126/science.abd9909 PMC7857403

[59] Alexpandi R , De Mesquita JF , Pandian SK and Ravi AV (2020) Quinolines‐based SARS‐CoV‐2 3CLpro and RdRp inhibitors and spike‐RBD‐ACE2 inhibitor for drug‐repurposing against COVID‐19: an in silico analysis. Front Microbiol 11, 1796. 32793181 10.3389/fmicb.2020.01796 PMC7390959

[60] Awad IE , Abu‐Saleh AAAA , Sharma S , Yadav A and Poirier RA (2020) High‐throughput virtual screening of drug databanks for potential inhibitors of SARS‐CoV‐2 spike glycoprotein. J Biomol Struct Dyn 40, 2099–2112. 33103586 10.1080/07391102.2020.1835721 PMC7643424

[61] Padhi AK , Seal A , Khan JM , Ahamed M and Tripathi T (2021) Unraveling the mechanism of arbidol binding and inhibition of SARS‐CoV‐2: insights from atomistic simulations. Eur J Pharmacol 894, 173836. 33387467 10.1016/j.ejphar.2020.173836 PMC7773528

[62] Kumar V , Liu H and Wu C (2021) Drug repurposing against SARS‐CoV‐2 receptor binding domain using ensemble‐based virtual screening and molecular dynamics simulations. Comput Biol Med 135, 104634. 34256255 10.1016/j.compbiomed.2021.104634 PMC8257406

[63] Wu K , Peng G , Wilken M , Geraghty RJ and Li F (2012) Mechanisms of host receptor adaptation by severe acute respiratory syndrome coronavirus. J Biol Chem 287, 8904–8911. 22291007 10.1074/jbc.M111.325803 PMC3308800

[64] Jawad B , Adhikari P , Podgornik R and Ching W‐Y (2021) Key interacting residues between RBD of SARS‐CoV‐2 and ACE2 receptor: combination of molecular dynamics simulation and density functional calculation. J Chem Inf Model 61, 4425–4441. 34428371 10.1021/acs.jcim.1c00560

[65] Verma J and Subbarao N (2021) Insilico study on the effect of SARS‐CoV‐2 RBD hotspot mutants' interaction with ACE2 to understand the binding affinity and stability. Virology 561, 107–116. 34217923 10.1016/j.virol.2021.06.009 PMC8237243

[66] Hsiao Y‐W , Bray DJ , Taddese T , Jiménez‐Serratos G and Crain J (2023) Structure adaptation in omicron SARS‐CoV‐2/hACE2: biophysical origins of evolutionary driving forces. Biophys J 122, 4057–4067. 37717145 10.1016/j.bpj.2023.09.003 PMC10624932

[67] Sergeeva AP , Katsamba PS , Liao J , Sampson JM , Bahna F , Mannepalli S , Morano NC , Shapiro L , Friesner RA and Honig B (2023) Free energy perturbation calculations of mutation effects on SARS‐CoV‐2 RBD::ACE2 binding affinity. J Mol Biol 435, 168187. 37355034 10.1016/j.jmb.2023.168187 PMC10286572

[68] Gong Y , Qin S , Dai L and Tian Z (2021) The glycosylation in SARS‐CoV‐2 and its receptor ACE2. Signal Transduct Target Ther 6, 396. 34782609 10.1038/s41392-021-00809-8 PMC8591162

[69] Watanabe Y , Bowden TA , Wilson IA and Crispin M (2019) Exploitation of glycosylation in enveloped virus pathobiology. Biochimica et Biophysica Acta (BBA)‐General Subjects 1863, 1480–1497. 31121217 10.1016/j.bbagen.2019.05.012 PMC6686077

[70] Watanabe Y , Berndsen ZT , Raghwani J , Seabright GE , Allen JD , Pybus OG , McLellan JS , Wilson IA , Bowden TA , Ward AB *et al*. (2020) Vulnerabilities in coronavirus glycan shields despite extensive glycosylation. Nat Commun 11, 2688. 32461612 10.1038/s41467-020-16567-0 PMC7253482

[71] Raman R , Tharakaraman K , Sasisekharan V and Sasisekharan R (2016) Glycan – protein interactions in viral pathogenesis. Curr Opin Struct Biol 40, 153–162. 27792989 10.1016/j.sbi.2016.10.003 PMC5526076

[72] Yang M , Huang J , Simon R , Wang L‐X and MacKerell AD Jr (2017) Conformational heterogeneity of the HIV envelope glycan shield. Sci Rep 7, 4435. 28667249 10.1038/s41598-017-04532-9 PMC5493700

[73] Crispin M , Ward AB and Wilson IA (2018) Structure and immune recognition of the HIV glycan shield. Annu Rev Biophys 47, 499–523. 29595997 10.1146/annurev-biophys-060414-034156 PMC6163090

[74] Watanabe Y , Allen JD , Wrapp D , McLellan JS and Crispin M (2020) Site‐specific glycan analysis of the SARS‐CoV‐2 spike. Science 369, 330–333. 32366695 10.1126/science.abb9983 PMC7199903

[75] Sun X , Jayaraman A , Maniprasad P , Raman R , Houser KV , Pappas C , Zeng H , Sasisekharan R , Katz JM and Tumpey TM (2013) N‐linked glycosylation of the hemagglutinin protein influences virulence and antigenicity of the 1918 pandemic and seasonal H1N1 influenza a viruses. J Virol 87, 8756–8766. 23740978 10.1128/JVI.00593-13 PMC3719814

[76] Alymova IV , York IA , Air GM , Cipollo JF , Gulati S , Baranovich T , Kumar A , Zeng H , Gansebom S and McCullers JA (2016) Glycosylation changes in the globular head of H3N2 influenza hemagglutinin modulate receptor binding without affecting virus virulence. Sci Rep 6, 36216. 27796371 10.1038/srep36216 PMC5086918

[77] Wang C‐C , Chen J‐R , Tseng Y‐C , Hsu C‐H , Hung Y‐F , Chen S‐W , Chen C‐M , Khoo K‐H , Cheng T‐J , Cheng Y‐SE *et al*. (2009) Glycans on influenza hemagglutinin affect receptor binding and immune response. Proc Natl Acad Sci USA 106, 18137–18142. 19822741 10.1073/pnas.0909696106 PMC2775302

[78] Kasson PM and Pande VS (2008) Structural basis for influence of viral Glycans on ligand binding by influenza hemagglutinin. Biophys J 95, L48–L50. 18641068 10.1529/biophysj.108.141507 PMC2547437

[79] Chen W , Sun S and Li Z (2012) Two glycosylation sites in H5N1 influenza virus hemagglutinin that affect binding preference by computer‐based analysis. PLoS One 7, e38794. 22719948 10.1371/journal.pone.0038794 PMC3375263

[80] Tekin ED (2023) Investigation of the effects of N‐Acetylglucosamine on the stability of the spike protein in SARS‐CoV‐2 by molecular dynamics simulations. Computational and Theoretical Chemistry 1222, 114049. 36743995 10.1016/j.comptc.2023.114049 PMC9890939

[81] Pandey VK , Sharma R , Prajapati GK , Mohanta TK and Mishra AK (2022) N‐glycosylation, a leading role in viral infection and immunity development. Mol Biol Rep 49, 8109–8120. 35364718 10.1007/s11033-022-07359-4 PMC8974804

[82] Singh JK , Singh J and Srivastava SK (2023) Investigating the role of glycans in omicron sub‐lineages XBB.1.5 and XBB.1.16 binding to host receptor using molecular dynamics and binding free energy calculations. J Comput Aided Mol Des 37, 551–563. 37542610 10.1007/s10822-023-00526-0

[83] Maity S and Acharya A (2024) Many roles of carbohydrates: a computational spotlight on the coronavirus S protein binding. ACS Appl Bio Mater 7, 646–656. 10.1021/acsabm.2c01064 PMC10880061 36947738

[84] Carbaugh DL and Lazear HM (2020) Flavivirus envelope protein glycosylation: impacts on viral infection and pathogenesis. J Virol 94, e00104. 32161171 10.1128/JVI.00104-20 PMC7269438

[85] Lan PD , Nissley DA , O'Brien EP , Nguyen TT and Li MS (2024) Deciphering the free energy landscapes of SARS‐CoV‐2 wild type and omicron variant interacting with human ACE2. J Chem Phys 160, 055101. 38310477 10.1063/5.0188053 PMC11223169

[86] Re S and Mizuguchi K (2021) Glycan cluster shielding and antibody epitopes on Lassa virus envelop protein. J Phys Chem B 125, 2089–2097. 33606939 10.1021/acs.jpcb.0c11516

[87] Seitz C , Deveci İ and McCammon JA (2023) Glycosylation and crowded membrane effects on influenza neuraminidase stability and dynamics. J Phys Chem Lett 14, 9926–9934. 37903229 10.1021/acs.jpclett.3c02524 PMC10641874

[88] Wood NT , Fadda E , Davis R , Grant OC , Martin JC , Woods RJ and Travers SA (2013) The influence of N‐linked glycans on the molecular dynamics of the HIV‐1 gp120 V3 loop. PLoS One 8, e80301. 24303005 10.1371/journal.pone.0080301 PMC3841175

[89] Stewart‐Jones GBE , Soto C , Lemmin T , Chuang G‐Y , Druz A , Kong R , Thomas PV , Wagh K , Zhou T , Behrens A‐J *et al*. (2016) Trimeric HIV‐1‐Env structures define glycan shields from clades a, B, and G. Cell 165, 813–826. 27114034 10.1016/j.cell.2016.04.010 PMC5543418

[90] Casalino L , Gaieb Z , Goldsmith JA , Hjorth CK , Dommer AC , Harbison AM , Fogarty CA , Barros EP , Taylor BC , Mclellan JS *et al*. (2020) Beyond shielding: the roles of glycans in the SARS‐CoV‐2 spike protein. ACS Central Science 6, 1722–1734. 33140034 10.1021/acscentsci.0c01056 PMC7523240

[91] Wrapp D , Wang N , Corbett KS , Goldsmith JA , Hsieh C‐L , Abiona O , Graham BS and McLellan JS (2020) Cryo‐EM structure of the 2019‐nCoV spike in the prefusion conformation. Science 367, 1260–1263. 32075877 10.1126/science.abb2507 PMC7164637

[92] Yuan Y , Cao D , Zhang Y , Ma J , Qi J , Wang Q , Lu G , Wu Y , Yan J , Shi Y *et al*. (2017) Cryo‐EM structures of MERS‐CoV and SARS‐CoV spike glycoproteins reveal the dynamic receptor binding domains. Nat Commun 8, 15092. 28393837 10.1038/ncomms15092 PMC5394239

[93] Gui M , Song W , Zhou H , Xu J , Chen S , Xiang Y and Wang X (2017) Cryo‐electron microscopy structures of the SARS‐CoV spike glycoprotein reveal a prerequisite conformational state for receptor binding. Cell Res 27, 119–129. 28008928 10.1038/cr.2016.152 PMC5223232

[94] Gur M , Taka E , Yilmaz SZ , Kilinc C , Aktas U and Golcuk M (2020) Conformational transition of SARS‐CoV‐2 spike glycoprotein between its closed and open states. J Chem Phys 153, 075101. 32828084 10.1063/5.0011141

[95] Valério M , Borges‐Araújo L , Melo MN , Lousa D and Soares CM (2022) SARS‐CoV‐2 variants impact RBD conformational dynamics and ACE2 accessibility. Frontiers in Medical Technology 4, 1009451. 36277437 10.3389/fmedt.2022.1009451 PMC9581196

[96] Melikyan GB (2008) Common principles and intermediates of viral protein‐mediated fusion: the HIV‐1 paradigm. Retrovirology 5, 111. 19077194 10.1186/1742-4690-5-111 PMC2633019

[97] Yao H and Hong M (2014) Conformation and lipid interaction of the fusion peptide of the paramyxovirus PIV5 in anionic and negative‐curvature membranes from solid‐state NMR. J Am Chem Soc 136, 2611–2624. 24428385 10.1021/ja4121956 PMC3985871

[98] Donald JE , Zhang Y , Fiorin G , Carnevale V , Slochower DR , Gai F , Klein ML and DeGrado WF (2011) Transmembrane orientation and possible role of the fusogenic peptide from parainfluenza virus 5 (PIV5) in promoting fusion. Proc Natl Acad Sci 108, 3958–3963. 21321234 10.1073/pnas.1019668108 PMC3054033

[99] Suárez T , Gómara MJ , Goñi FM , Mingarro I , Muga A , Pérez‐Payá E and Nieva JL (2003) Calcium‐dependent conformational changes of membrane‐bound Ebola fusion peptide drive vesicle fusion. FEBS Lett 535, 23–28. 12560072 10.1016/s0014-5793(02)03847-4

[100] White SH and Wimley WC (1999) Membrane protein folding and stability: physical principles. Annu Rev Biophys Biomol Struct 28, 319–365. 10410805 10.1146/annurev.biophys.28.1.319

[101] Kielian M , Klimjack MR , Ghosh S and Duffus WA (1996) Mechanisms of mutations inhibiting fusion and infection by Semliki Forest virus. J Cell Biol 134, 863–872. 8769412 10.1083/jcb.134.4.863 PMC2120960

[102] Lousa D , Pinto ART , Victor BL , Laio A , Veiga AS , Castanho MA and Soares CM (2016) Fusing simulation and experiment: the effect of mutations on the structure and activity of the influenza fusion peptide. Sci Rep 6, 1–14. 27302370 10.1038/srep28099 PMC4908596

[103] Yao H and Hong M (2013) Membrane‐dependent conformation, dynamics, and lipid interactions of the fusion peptide of the paramyxovirus PIV5 from solid‐state NMR. J Mol Biol 425, 563–576. 23183373 10.1016/j.jmb.2012.11.027 PMC4082994

[104] Lai AL , Moorthy AE , Li Y and Tamm LK (2012) Fusion activity of HIV gp41 fusion domain is related to its secondary structure and depth of membrane insertion in a cholesterol‐dependent fashion. J Mol Biol 418, 3–15. 22343048 10.1016/j.jmb.2012.02.010 PMC3654243

[105] Schaefer SL , Jung H and Hummer G (2021) Binding of SARS‐CoV‐2 fusion peptide to host endosome and plasma membrane. J Phys Chem B 125, 7732–7741. 34255499 10.1021/acs.jpcb.1c04176 PMC8311640

[106] Han X , Bushweller JH , Cafiso DS and Tamm LK (2001) Membrane structure and fusion‐triggering conformational change of the fusion domain from influenza hemagglutinin. Nat Struct Biol 8, 715–720. 11473264 10.1038/90434

[107] Lorieau JL , Louis JM and Bax A (2010) The complete influenza hemagglutinin fusion domain adopts a tight helical hairpin arrangement at the lipid: water interface. Proc Natl Acad Sci 107, 11341–11346. 20534508 10.1073/pnas.1006142107 PMC2895095

[108] Cross KJ , Langley WA , Russell RJ , Skehel JJ and Steinhauer DA (2009) Composition and functions of the influenza fusion peptide. Protein Pept Lett 16, 766–778. 19601906 10.2174/092986609788681715

[109] Huang Q , Chen C‐L and Herrmann A (2004) Bilayer conformation of fusion peptide of influenza virus hemagglutinin: a molecular dynamics simulation study. Biophys J 87, 14–22. 15240440 10.1529/biophysj.103.024562 PMC1304337

[110] Li J , Das P and Zhou R (2010) Single mutation effects on conformational change and membrane deformation of influenza hemagglutinin fusion peptides. J Phys Chem B 114, 8799–8806. 20552971 10.1021/jp1029163

[111] Légaré S and Lagüe P (2012) The influenza fusion peptide adopts a flexible flat V conformation in membranes. Biophys J 102, 2270–2278. 22677380 10.1016/j.bpj.2012.04.003 PMC3353013

[112] Légaré S and Lagüe P (2014) The influenza fusion peptide promotes lipid polar head intrusion through hydrogen bonding with phosphates and N‐terminal membrane insertion depth. Proteins: Struct Funct Bioinf 82, 2118–2127. 10.1002/prot.24568 24668589

[113] Victor BL , Lousa D , Antunes JM and Soares CM (2015) Self‐assembly molecular dynamics simulations shed light into the interaction of the influenza fusion peptide with a membrane bilayer. J Chem Inf Model 55, 795–805. 25826469 10.1021/ci500756v

[114] Worch R , Filipek A , Krupa J , Szymaniec A and Setny P (2017) Three conserved residues of influenza fusion peptide alter its behavior at the membrane interface. Eur Biophys J Biophys Lett 233, 392. 10.1016/j.bbagen.2016.11.004 27825831

[115] Worch R , Dudek A , Krupa J , Szymaniec A and Setny P (2018) Charged N‐terminus of influenza fusion peptide facilitates membrane fusion. Int J Mol Sci 19, 578. 29443945 10.3390/ijms19020578 PMC5855800

[116] Larsson P and Kasson PM (2013) Lipid tail protrusion in simulations predicts fusogenic activity of influenza fusion peptide mutants and conformational models. PLoS Comput Biol 9, e1002950. 23505359 10.1371/journal.pcbi.1002950 PMC3591293

[117] Zhang Y , Ghosh U , Xie L , Holmes D , Severin KG and Weliky DP (2023) Lipid acyl chain protrusion induced by the influenza virus hemagglutinin fusion peptide detected by NMR paramagnetic relaxation enhancement. Biophys Chem 299, 107028. 37247572 10.1016/j.bpc.2023.107028 PMC10330521

[118] Kasson PM , Lindahl E and Pande VS (2010) Atomic‐resolution simulations predict a transition state for vesicle fusion defined by contact of a few lipid tails. PLoS Comput Biol 6, e1000829. 20585620 10.1371/journal.pcbi.1000829 PMC2891707

[119] Valério M , Mendonça DA , Morais J , Buga CC , Cruz CH , Castanho MARB , Melo MN , Soares CM , Veiga AS and Lousa D (2022) Parainfluenza fusion peptide promotes membrane fusion by assembling into Oligomeric Porelike structures. ACS Chem Biol 17, 1831–1843. 35500279 10.1021/acschembio.2c00208 PMC9295702

[120] Wang W , Tan J and Ye S (2020) Unsaturated lipid accelerates formation of Oligomeric β‐sheet structure of GP41 fusion peptide in model cell membrane. J Phys Chem B 124, 5169–5176. 32453953 10.1021/acs.jpcb.0c02464

[121] Meher G , Sinha S , Pattnaik GP , Dastidar SG and Chakraborty H (2019) Cholesterol modulates membrane properties and the interaction of gp41 fusion peptide to promote membrane fusion. J Phys Chem B 33, 7113–7122. 10.1021/acs.jpcb.9b04577 31345037

[122] Villalaín J (2022) Envelope E protein of dengue virus and phospholipid binding to the late endosomal membrane. Biochim Biophys Acta Biomembr 1864, 183889. 35167815 10.1016/j.bbamem.2022.183889

[123] Campbell O and Monje‐Galvan V (2023) Lipid composition modulates interactions of p7 viroporin during membrane insertion. J Struct Biol 215, 108013. 37586469 10.1016/j.jsb.2023.108013

[124] Hao Y , Wu R , Wang F , Zhang L , Wang Z , Song X and Liu L (2022) Functional peptides from SARS‐CoV‐2 binding with cell membrane: from molecular dynamics simulations to cell demonstration. Cells 11, 1738. 35681433 10.3390/cells11111738 PMC9179371

[125] Lynch DL , Pavlova A , Fan Z and Gumbart JC (2023) Understanding virus structure and dynamics through molecular simulations. J Chem Theory Comput 19, 3025–3036. 37192279 10.1021/acs.jctc.3c00116 PMC10269348

[126] Soñora M , Barrera EE and Pantano S (2022) The stressed life of a lipid in the Zika virus membrane. Biochim Biophys Acta Biomembr 1864, 183804. 34656553 10.1016/j.bbamem.2021.183804

[127] White JM , Ward AE , Odongo L and Tamm LK (2023) Viral membrane fusion: a dance between proteins and lipids. Annual Review of Virology 10, 139–161. 10.1146/annurev-virology-111821-093413 PMC10866366 37774128

[128] Van Doren SR , Scott BS and Koppisetti RK (2023) SARS‐CoV‐2 fusion peptide sculpting of a membrane with insertion of charged and polar groups. Structure 31, 1184–1199.e3. 37625399 10.1016/j.str.2023.07.015 PMC10592393

[129] Nilsson LB , Sun F , Kadupitiya JCS and Jadhao V (2023) Molecular dynamics simulations of deformable viral Capsomers. Viruses 15, 1672. 37632014 10.3390/v15081672 PMC10459744

[130] Rice A , Haldar S , Wang E , Blank PS , Akimov SA , Galimzyanov TR , Pastor RW and Zimmerberg J (2022) Planar aggregation of the influenza viral fusion peptide alters membrane structure and hydration, promoting poration. Nat Commun 13, 1–15. 36470871 10.1038/s41467-022-34576-z PMC9722698

[131] Shen H and Wu Z (2022) Effect of disulfide bridge on the binding of SARS‐CoV‐2 fusion peptide to cell membrane: a coarse‐grained study. ACS Omega 7, 36762–36775. 36278087 10.1021/acsomega.2c05079 PMC9583636

[132] Khelashvili G , Plante A , Doktorova M and Weinstein H (2021) Ca^2+^−dependent mechanism of membrane insertion and destabilization by the SARS‐CoV‐2 fusion peptide. Biophys J 120, 1105–1119. 33631204 10.1016/j.bpj.2021.02.023 PMC7899928

[133] Pabis A , Rawle RJ and Kasson PM (2020) Influenza hemagglutinin drives viral entry via two sequential intramembrane mechanisms. Proc Natl Acad Sci 117, 7200–7207. 32188780 10.1073/pnas.1914188117 PMC7132276

[134] Cross KJ , Wharton SA , Skehel JJ , Wiley DC and Steinhauer DA (2001) Studies on influenza haemagglutinin fusion peptide mutants generated by reverse genetics. EMBO J 20, 4432–4442. 11500371 10.1093/emboj/20.16.4432 PMC125568

[135] Su R , Zeng J , Marcink TC , Porotto M , Moscona A and O'Shaughnessy B (2023) Host cell membrane capture by the SARS‐CoV‐2 spike protein fusion intermediate. ACS Cent Sci 9, 1213–1228. 37396856 10.1021/acscentsci.3c00158 PMC10255576

[136] Xu D and Zhang Y (2012) Ab initio protein structure assembly using continuous structure fragments and optimized knowledge‐based force field. Proteins: Struct Funct Bioinf 80, 1715–1735. 10.1002/prot.24065 PMC3370074 22411565

[137] Hunt AC , Case JB , Park Y‐J , Cao L , Wu K , Walls AC , Liu Z , Bowen JE , Yeh H‐W , Saini S *et al*. (2022) Multivalent designed proteins neutralize SARS‐CoV‐2 variants of concern and confer protection against infection in mice. Sci Transl Med 14, eabn1252. 35412328 10.1126/scitranslmed.abn1252 PMC9258422

[138] Humphrey W , Dalke A and Schulten K (1996) VMD: Visual molecular dynamics. J Mol Graph 14, 33–38. 8744570 10.1016/0263-7855(96)00018-5

[139] Welch BD , Liu Y , Kors CA , Leser GP , Jardetzky TS and Lamb RA (2012) Structure of the cleavage‐activated prefusion form of the parainfluenza virus 5 fusion protein. Proc Natl Acad Sci 109, 16672–16677. 23012473 10.1073/pnas.1213802109 PMC3478641

[140] Lousa D and Soares CM (2021) Molecular mechanisms of the influenza fusion peptide: insights from experimental and simulation studies. FEBS Open Bio 11, 2211–5463. 10.1002/2211-5463.13323 PMC8634857 34710289

